# Supplementary material for: Differential Gene Expression Pattern of Importin β3 and NS5 in C6/36 Cells Acutely and Persistently Infected with Dengue Virus 2
Source: Pathogens. 2023 Jan 27;12(2):191. doi: 10.3390/pathogens12020191 (PMC9966734; doi:10.3390/pathogens12020191)
Supplement: Supplementary file 1 [file pathogens-12-00191-s001.zip › Supplementary figures.pdf]

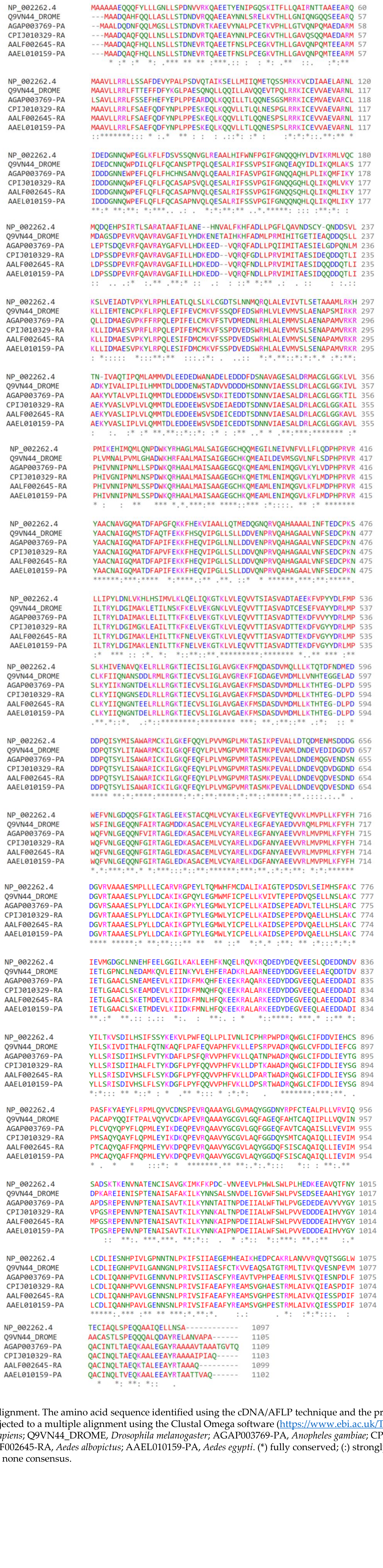

Figure S1. Multiple alignment. The amino acid sequence identified using the cDNA/AFLP technique and the primers ES4/MS4 (XM 001654246.2) was subjected to a multiple alignment using the Clustal Omega software (https://www.ebi.ac.uk/Tools/msa/clustalo/). NP_002262.4, *Homo sapiens*; Q9VN44_DROME, *Drosophila melanogaster*; AGAP003769-PA, *Anopheles gambiae*; CPIJ010329-RA, *Culex quinquefasciatus*; AALF002645-RA, *Aedes albopictus*; AAEL010159-PA, *Aedes egypti*. (*) fully conserved; (:) strongly conserved; (.) weakly conserved; (-) none consensus.

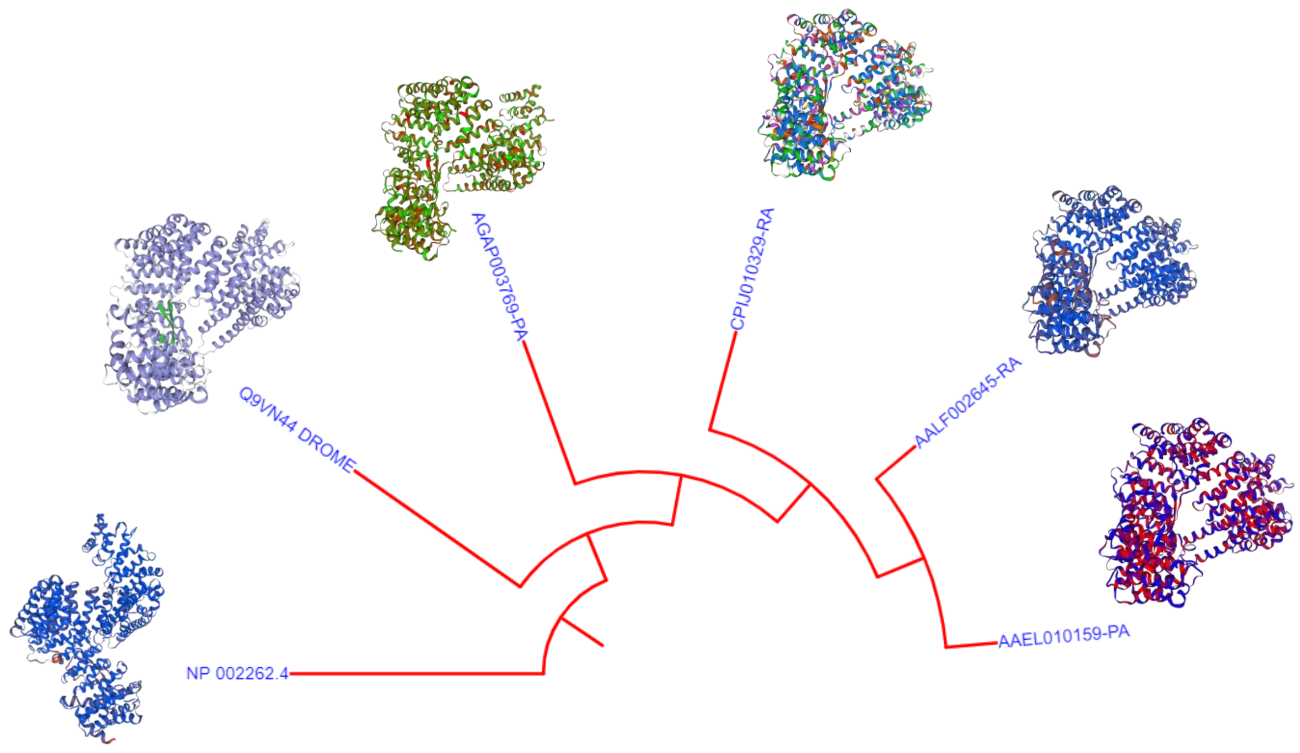

Figure S2. Phylogenetic analysis. The amino acid sequences of proteins identified by alignment with importin 5 were subjected to iTOL software (https://itol.embl.de/upload.cgi). The structure prediction was performed using SWISS-MODEL software (https://swissmodel.expasy.org). NP_002262.4, *Homo sapiens*; Q9VN44_DROME, *Drosophila melanogaster*; AGAP003769-PA, *Anopheles gambiae*; CPIJ010329-RA, *Culex quinquefasciatus*; AALF002645-RA, *Aedes albopictus*; AAEL010159-PA, *Aedes egypti*.

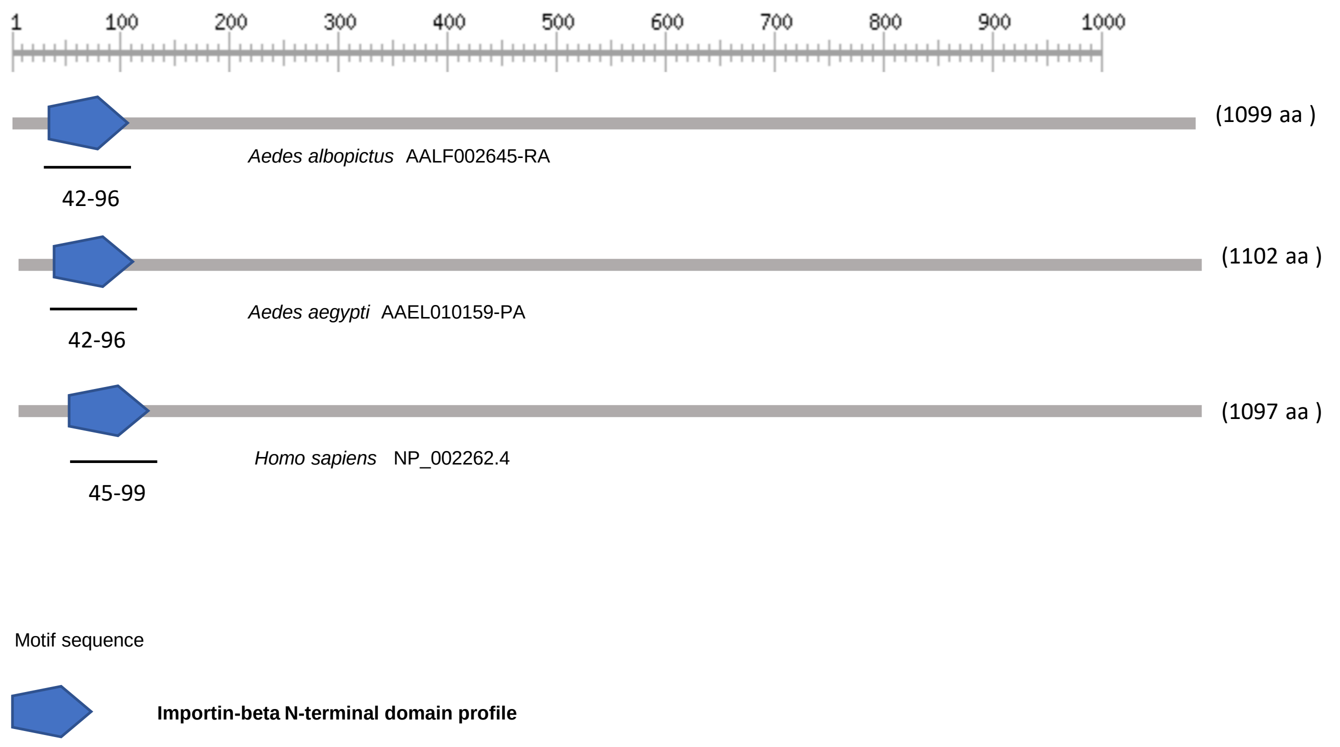

Clustal Omega aligment of motif sequence

```
AALF002645-RA      LLGAVQNPQMTEEARMMAAVLLRRLFSAEFQDFYNPLPPESKEQLKQQVLLTLQQ      55
AAEL010159-PA      LLGAVQNPQMTEEARMMAAVLLRRLFSAEFQDFYNPLPPESKEQLKQQVLLTLQQ      55
NP_002262.4        LLQAIRNTTAAEEARQMAAVLLRRLLSSAFDEVYPALPSDVQTAIKSELLMIIQM      55
                   ** *::*   :**** *********:*: *:..*  ** : :  :*.::*: :*
```

Figure S3. Importin β motif**.** The N-terminal importin β motif (lower panel) was located in impotins of *Homo sapiens* and *Aedes* mosquitoes using PROSITE software.

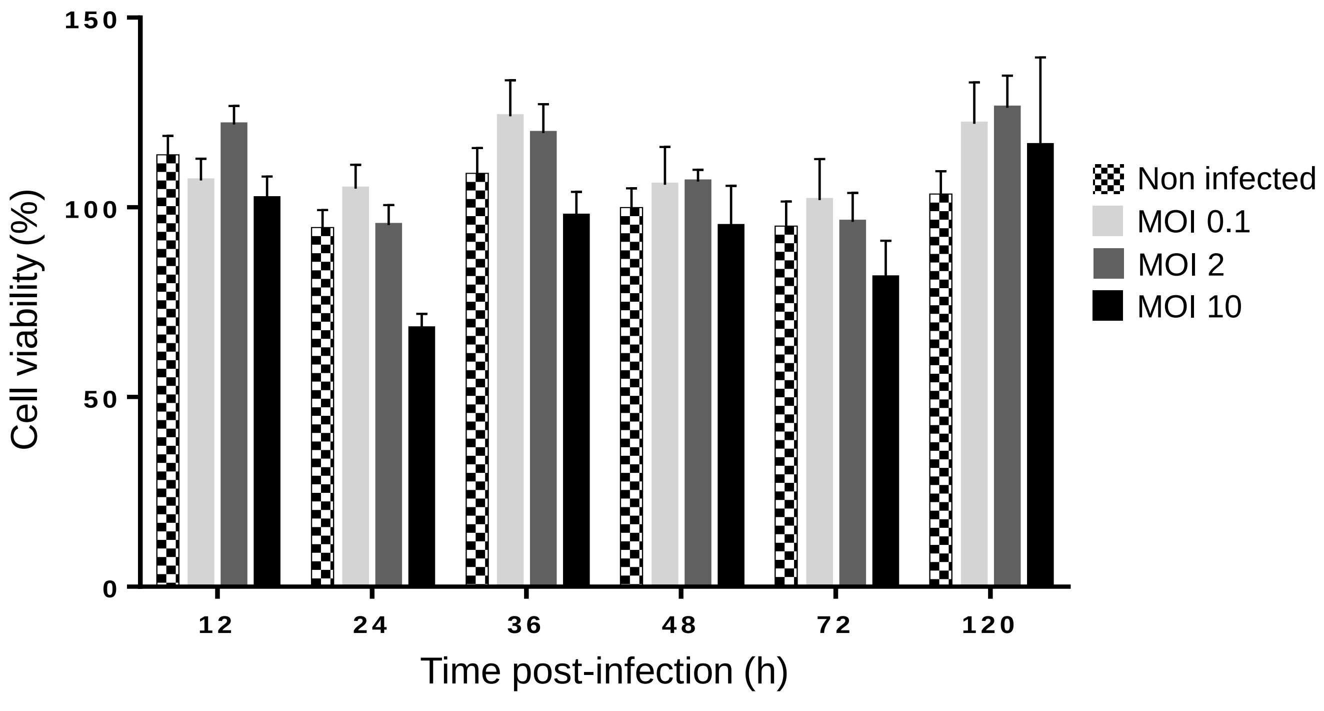

Figure S4. Cell viability analysis in C6/36 cells at different MOIs and time points. Cell viability in DENV-2 infected C6/36 at an MOI of 0.1, 2 and 10 at 12, 24, 36, 48, 72 and 120 hours and mock-infected C6/36 (Non-infected) cells was determined by a MTT assay. Results were expressed in percentage as mean ± SEM from four independent assays. Two way ANOVA and multiple comparisons test of Tukey were performed for statistical analysis of the data.
