# Supplementary material for: Differential Gene Expression Pattern of Importin β3 and NS5 in C6/36 Cells Acutely and Persistently Infected with Dengue Virus 2
Source: Pathogens. 2023 Jan 27;12(2):191. doi: 10.3390/pathogens12020191 (PMC9966734; doi:10.3390/pathogens12020191)
Supplement: Supplementary file 1 [file pathogens-12-00191-s001.zip › Supplementary table S1.pdf]

Table S1. Sequence of the primers used.

Table S1.1. Adaptors used for cDNA-AFLP technique

Adaptor	Sequence
**EcoRI adaptor**	
Leader strand	5′ CTCGTAGACTGCGTACC 3′
Complementary strand	5′ AATTGGTACGCAGTCTAC 3′
**MseI adaptor**	
Leader strand	5′ GACGATGAGTCCTGAG 3′
Complementary strand	5′ TACTCAGGACTCAT 3′

Table S1.2. Primers used for pre-selective PCR amplification

Primer	Sequence
**Pre-selective EcoRI primer**	
E1	5′ GACTGCGTACCAATTC 3′
**Pre-selective MseI primer**	
M1	5′ GATGAGTCCTGAGTAA 3′

Table S1.3. Primers used for selective PCR amplification

Primer	Sequence
**Selective EcoRI primers**	
ES1	5′ GACTGCGTACCAATTCACA 3′
ES2	5′ GACTGCGTACCAATTCAAC 3′
ES3	5′ GACTGCGTACCAATTCAAG 3′
ES4	5′ GACTGCGTACCAATTCACC 3′
ES5	5′ GACTGCGTACCAATTCACG 3′
ES6	5′ GACTGCGTACCAATTCACT 3′
ES7	5′ GACTGCGTACCAATTCAGG 3′
**Selective MseI primers**	
MS1	5′ GATGAGTCCTGAGTAACAC 3′
MS2	5′ GATGAGTCCTGAGTAACAT 3′
MS3	5′ GATGAGTCCTGAGTAACAA 3′
MS4	5′ GATGAGTCCTGAGTAACAG 3′
MS5	5′ GATGAGTCCTGAGTAACTC 3′
MS6	5′ GATGAGTCCTGAGTAACTA 3′
MS7	5′ GATGAGTCCTGAGTAACGA 3′
MS8	5′ GATGAGTCCTGAGTAACGT 3′
MS9	5′ GATGAGTCCTGAGTAACCG 3′
MS10	5′ GATGAGTCCTGAGTAATAA 3′
MS11	5′ GATGAGTCCTGAGTAATAG 3′
MS12	5′ GATGAGTCCTGAGTAATTA 3′
MS13	5′ GATGAGTCCTGAGTAATCA 3′

Table S1.4. Primers used for qPCR amplification

Gene	Type	Sequences (5´-3´)
Importin-β3	Forward	CGACAACGATTGGCAGTTTG
Importin-β3	Reverse	GACTCCGTCATGGAAGTAGAAC
Importin-β3	TaqMan Probe	FAM/ ATTCGCCAACTACGCCGAAGAAGT /BHQ1
S7 rRNA	Forward	AAC AAG CAG AAG CGT CCA
S7 rRNA	Reverse	GTA CAC TGA CGT GAA GGT GTC
S7 rRNA	TaqMan Probe	FAM/ ATG GTG GTC TGC TGG TTC TTG TCC /BHQ1
