# Supplementary material for: Differential Gene Expression Pattern of Importin β3 and NS5 in C6/36 Cells Acutely and Persistently Infected with Dengue Virus 2
Source: Pathogens. 2023 Jan 27;12(2):191. doi: 10.3390/pathogens12020191 (PMC9966734; doi:10.3390/pathogens12020191)
Supplement: Supplementary file 1 [file pathogens-12-00191-s001.zip › Supplementary table S2.pdf]

Table S2. Identification of the amplicons

Combination	Condition	Gene	Accession number
ES4-MS4	NI	*Aedes aegypti* importin 5	XM 001654246.2
ES1-MS10	NI	*Aedes aegypti* molybdopterin cofactor synthesis protein	AAEL000126
		Aedes aegypti ethanolamine-phosphate cytidyltransferase	AAEL005651
ES1-MS11	NI	*Aedes aegypti* ASAP ID:43290 conserved unknown mRNA sequence	AY431727.1
		Aedes aegypti hypothetical protein	AAEL002467
		Aedes aegypti peritrophin	AY050566.1
		Aedes aegypti clone BAC ND41C6	EF173374.1
		Aedes aegypti ferritin subunit	AAEL010393
		Aedes aegypti adenylate cyclase	AAEL005177
		Aedes aegypti hypothetical protein	AAEL009751
		Aedes aegypti clone BAC ND67B23	EF173378.1
ES4-MS6	PI	*Aedes aegypti* hypothetical protein	AAEL008802
		Aedes aegypti clone AE-289 putative salivary secreted peptide	DQ440046.1
		Aedes aegypti ASAP ID:42879 conserved unknown mRNA	AY433328.1
ES3-MS9	PI	*Aedes aegypti* inosine-5-monophosphate dehydrogenase	XM C01653725.2
		Aedes albopictus clone AL 133 ribosomal protein S8	AY826137.1
		Aedes aegypti 40S ribosomal protein S8	XM 001653071.2
		Aedes aegypti clone AE-266 40S ribosomal protein S8	DQ440035.1
		Aedes aegypti ASAP ID:36093 cytosolic small ribosomal subunit S8	AY432409.1
		Single read from an extremity of al full-length cDNA clone made form *Aedes aegypti* total adult females 5′ end of clone KW0AAA1YG07AAM1 from strain Liverpool	CR938578.1
		Single read from an extremity of al full-length cDNA clone made form *Aedes aegypti* total adult females 3′ end of clone KW0AAA1YG07BBM1 from strain Liverpool	CR938577.1
		Aedes aegypti clone BAC ND41B18	EF173373.1

Combination: oligonucleotides used for selective PCR amplification
Condition: NI, non-infected cells. PI, persistently infected cells
Accession number: According with GeneBank database (NCBI)
